# Muscle quality as a complementary prognostic tool in conjunction with sarcopenia assessment in younger and older individuals

**DOI:** 10.1007/s00421-019-04107-8

**Published:** 2019-02-26

**Authors:** Matthew J. Lees, Oliver J. Wilson, Karen Hind, Theocharis Ispoglou

**Affiliations:** 1 Institute for Sport, Physical Activity and Leisure, Leeds Beckett University, Fairfax Hall, Headingley Campus, Leeds, West Yorkshire LS6 3QS UK; 2 Department of Sport and Exercise Sciences, Durham University, Durham, UK

**Keywords:** Muscle mass, Dynapenia, Functional capacity, Strength, Ageing

## Abstract

**Purpose:**

This pilot study investigated differences in lean tissue mass, muscle strength, muscle quality (strength per unit of muscle mass; MQ), and functional performance in healthy younger and older individuals. The most robust predictors of appendicular lean mass (ALM) were then determined in each group.

**Methods:**

Fifty younger (18–45 years) and 50 older (60–80 years) participants completed tests of upper and lower body strength alongside body composition by dual-energy X-ray absorptiometry from which upper- and lower-body MQ were estimated. Available cut-points for older people were used to determine low upper-body MQ in both groups. Low lower-body MQ was determined as at least two standard deviations below the mean of the younger group. Functional performance was assessed by gait speed. Sarcopenia was identified using two established definitions.

**Results:**

Upper and lower body strength, ALM, lower-body MQ and gait speed were significantly higher in the younger group (all *p* < 0.002). Sarcopenia was identified in 2–4% of the older group. Low upper-body MQ was evident in 32% and 42% of the younger and older group, respectively. Low lower-body MQ was observed in 4% of younger participants, and 50% of older participants. In both groups, the most robust predictors of ALM were upper and lower body strength (young *R*^2^ = 0.74, 0.82; older *R*^2^ = 0.68, 0.72).

**Conclusions:**

Low MQ despite low prevalence rates of sarcopenia in both groups suggests a need for age-specific MQ cut-points. Muscle quality assessments might be useful complementary prognostic tools alongside existing sarcopenia definitions.

## Introduction

The progressive loss of skeletal muscle mass and strength with age is associated with a number of adverse health outcomes, such as decreased quality of life, functional impairment, disability, increased risk of falls, hospitalisation, and mortality (Batsis et al. 2015; Beaudart et al. 2017; Lauretani et al. 2003; Bischoff-Ferrari et al. 2015; Cesari et al. 2009; Gariballa and Alessa 2013; Guralnik et al. 2000; Landi et al. 2013). The early identification of individuals with low muscle mass and impaired physical function may promote desirable patient outcomes over the long-term. The term *sarcopenia* was first used to describe the age-associated decline in skeletal muscle mass (Rosenberg 1989). This definition has expanded to include measures of both skeletal muscle mass and function to determine the presence of sarcopenia (Cruz-Jentoft et al. 2010; Goodpaster et al. 2006). The absence of a consensus definition for sarcopenia represents a considerable challenge for assessing its prevalence and impact on public health (Beaudart et al. 2014; Mayhew et al. 2018; Batsis et al. 2015). Importantly, the prevalence of sarcopenia appears to be greater when assessing muscle mass only (24.2–40.4%) as opposed to measures of muscle mass, strength and/or physical function in tandem (9.9–18.6%) (Mayhew et al. 2018).

Muscle quality (MQ), typically defined as muscle strength or power per unit of muscle mass (Barbat-Artigas et al. 2012), is a key determinant of muscle function in later life (McGregor et al. 2014), and declines with age (Newman et al. 2003). A number of factors may mediate this, such as a decrease in the number and cross-sectional area of type II fibres, fat infiltration, and neurological derangements (McGregor et al. 2014; Fragala et al. 2015). Therefore, the inclusion of MQ assessment to augment existing sarcopenia definitions might serve as a more effective means of identifying individuals at risk of impaired mobility in later life (Cruz-Jentoft et al. 2018). Paradoxically, cut-points for handgrip strength have been lowered by the European Working Group on Sarcopenia in Older People (EWGSOP) (Cruz-Jentoft et al. 2018) in comparison with previous recommendations (Cruz-Jentoft et al. 2010). This reduces emphasis on MQ, since strength is a component of MQ determination (Barbat-Artigas et al. 2012).

In addition to assessing upper body strength, the assessment of lower body strength and function in older adults is important, since age-related declines are faster in lower body than upper body strength (Goodpaster et al. 2006; Janssen et al. 2000). Incorporation of upper and lower body strength is preferential to upper body strength alone (Yeung et al. 2018). In addition, low knee extension strength is a better predictor of mortality risk than low handgrip strength (Mitchell et al. 2012; Laukkanen et al. 1995), and handgrip strength alone does not provide a good measure to evaluate the effectiveness of interventions in frail populations (Tieland et al. 2015).

Methods employed to assess lower body strength are varied, whereby isokinetic dynamometry (Krause et al. 2012; Newman et al. 2006; Bouchard et al. 2011), a force transducer (Yeung et al. 2018), spring gauge (Hairi et al. 2010), and adopted proxy measures (Cruz-Jentoft et al. 2018) have been used. Isokinetic dynamometry is often considered the ‘gold standard’ for lower-body strength testing and has been used consistently in studies with older populations (Lauretani et al. 2003; Brach et al. 2004; Newman et al. 2003, 2006; Goodpaster et al. 2006; Scott et al. 2014). The availability of such costly and large-scale equipment to assess lower body strength might be restricted in clinical and epidemiological settings (Martin et al. 2006; Yeung et al. 2018). Therefore, the use of practical exercise training-specific equipment to establish leg extension strength might be a more cost-effective and accessible alternative to isokinetic dynamometry. For instance, leg extension one-repetition maximum (1RM) strength is strongly associated with peak torque values obtained using isokinetic dynamometry (*r* = 0.78–0.88), and to a greater extent than a leg press 1RM test (*r* = 0.72–0.77) (Verdijk et al. 2009). The need for lower-body strength testing as part of the comprehensive geriatric assessment was highlighted by Yeung et al. (2018), who demonstrated stronger associations of health characteristics with knee extension strength in comparison with handgrip strength in community-dwelling older adults. All of the above emphasise the need to assess lower body strength and muscle quality in older populations as a means to identify individuals at risk of impaired physical function and/or sarcopenia.

The objectives of this pilot study were to investigate differences in lean tissue mass, muscle strength, functional performance, and physical function among healthy young and older individuals and establish the relationships of these variables with total appendicular lean mass (ALM). We then sought to assess the presence of sarcopenia using two established definitions with the lowest and highest pooled prevalence estimates (Mayhew et al. 2018). We also performed MQ assessment to identify individuals with evidence of functional decline and aimed to reconcile these findings with sarcopenia definitions.

## Materials and methods

### Study design

A cross-sectional study involving two testing sessions, separated by a minimum period of 7 days but no longer than 14 days, was conducted between October 2016 and July 2017. In the first visit, measures of strength, body composition, functional performance and physical activity were taken and participants were familiarised with the lower body 1RM test. On the second visit, participants completed the 1RM test only. All procedures were conducted by the same trained researcher at the same time of day, under strict controls to mitigate the impact of confounding variables such as exercise and diet.

### Participants

Fifty younger (18–45 years; 30 males, 20 females) and 50 older (60–80 years; 24 males, 26 females) participants were randomly recruited from local universities and the wider community, using a combination of email communications, online or printed advertisements and leaflets. Participant characteristics for both groups are provided in Table 1. All participants were independently living and free from cardiovascular, metabolic, endocrine, or other metabolic disease. Participants were excluded if they smoked, used oestrogens within the previous 3 months, had a pacemaker, or were taking any medication known to affect protein metabolism (i.e., anabolic steroids, corticosteroids, and peripheral vasodilators). Younger females were excluded if they were using the oral contraceptive pill. Participants were randomly recruited from a range of sports teams and activity groups, as well as from the lay public, to represent a broad range of physical activity levels. Written informed consent was provided prior to commencing the study and all procedures were carried out in accordance with the Helsinki Declaration of 1964, following approval by the University Faculty Research Ethics Committee.

**Table 1:** Participant characteristics and baseline screening for the sample population (*n* = 100)

Variable	Young (*n* = 50)	Older (*n* = 50)	*P* value
Age (years)	23 ± 5	70 ± 4	< 0.0001
Height (cm)	174.8 ± 8.6	166.3 ± 10.1	< 0.0001
Body mass (kg)	74.4 ± 12.0	70.9 ± 15.2	0.202
BMI (kg/m^2^)	24.2 ± 2.6	25.4 ± 3.7	0.062
RHR (bpm)	64 ± 12	61 ± 10	0.125
SBP (mmHg)	114 ± 10	128 ± 12	< 0.0001
DBP (mmHg)	77 ± 15	80 ± 11	0.312

Data presented as mean ± SD

*BMI* body mass index, *DBP* diastolic blood pressure, *RHR* resting heart rate, *SBP* systolic blood pressure

### Preliminary screening and basic anthropometry

Participants arrived at the laboratory between 07.00 and 09.00 in a fasted, euhydrated state after avoiding alcohol and exercise for a 24 h period. Following an initial briefing, resting heart rate (RHR), systolic and diastolic blood pressure (SBP and DBP, respectively) were measured using a manual sphygmomanometer (Accoson Greenlight 300, Accoson, United Kingdom). Stature (to the nearest 0.1 cm) and body mass (to the nearest 0.1 kg) were recorded using a stadiometer (Seca 220, Hamburg, Germany) and calibrated electronic scales (Seca 220, Hamburg, Germany). Body mass index (BMI) was derived by calculating mass/height^2^. Finger capillary blood samples were obtained and analysed for total cholesterol, high-density lipoprotein, low-density lipoprotein, triglycerides and glucose using a Cholestech LDX (Alere San Diego, Inc., San Diego, CA) capillary whole blood lipid analyser (Donato et al. 2015).

### Body composition assessment

Total-body fat mass, lean tissue mass, bone mineral content and percentage tissue fat mass (%TFM) values were ascertained by one total-body dual-energy X-ray absorptiometry (DXA) scan (GE Lunar iDXA, GE Healthcare, Madison, WI). These data were used to calculate appendicular lean mass (ALM). Participants were scanned in a fasted, euhydrated state as per established recommendations (Nana et al. 2012; Sawka et al. 2007). Participants removed shoes and jewellery before receiving the scan, whilst adopting a supine position with arms to the side in the semi-prone position (Thurlow et al. 2018) and ankles supported with the Lunar ankle strap (0.5 cm space between the ankles). Standard mode scans took approximately 7.5 min to complete, whereas those > 100 kg in body mass (*n* = 2) necessitated the use of the thick mode scan, which took approximately 12.5 min.

The values for the body composition outcomes were determined from the ratio of soft tissue attenuation of two X-ray energy beams for each pixel containing a minimal amount of soft tissue but no significant bone (Mazess et al. 1990). In our laboratory, the in vivo short-term precision (%CV) for total-body composition variables are 0.82% for fat mass, 0.51% for lean mass, 0.86% for percentage body fat and 0.60% for bone mineral content (Hind et al. 2011). The machine was checked and calibrated on a daily basis in line with the manufacturer’s recommendations. All scanning and analysis procedures were performed by the same trained operator using the Lunar enCORE software package (version 15.0).

### Functional performance and strength testing

Gait speed was measured directly using a 6-m course and photoelectric timing gates (Witty, Microgate, Bolzano, Italy). Participants were instructed to walk at their usual pace between two gates, with the fastest of these attempts used for the final analysis. The established cut-point of < 1 m/s was used to characterise impaired physical performance (Cesari et al. 2005, 2009; Visser et al. 2002).

Dominant and non-dominant handgrip strength (specific force, kg) were assessed using a Takei T.K.K. 5401 GRIP-D handgrip dynamometer (Takei Scientific Instruments Co., Ltd, Niigata, Japan), with the participant in a seated position (knees at 90°) and the shoulders adducted and neutrally rotated. The elbow was flexed at 90° with the forearm in a neutral position and the wrist in slight extension (0°–30°) (De Dobbeleer et al. 2017). The participant was then asked to squeeze the handle of the dynamometer as hard as possible for a maximum of five seconds. The highest of three attempts was noted and the process was repeated for the non-dominant hand. The previously established cut-points of < 30 kg for males and < 20 kg for females, respectively, were used for the determination of low grip strength (Lauretani et al. 2003).

For testing of lower body strength, participants were asked to perform separate 1RM tests of the dominant and non-dominant limbs using a Cybex VRS Prestige (Cybex International, Medway, MA) leg extension machine. A 5 min dynamic warmup was performed, with emphasis on the lower body musculature, prior to beginning a protocol-specific warm-up. Each participant’s 1RM was measured using previously described procedures (Baechle and Earle 2008). At the initial laboratory visit, participants completed a 1RM test for familiarisation purposes which was then repeated on the subsequent visit to establish 1RM.

### Assessment of habitual physical activity

Participants were asked to provide estimates of physical activity over the previous 7 days, using the long form of the International Physical Activity Questionnaire (IPAQ). The estimated total time spent in the respective domains of occupational, transport, household and leisure-related physical activity were calculated. These data were then used to determine weighted MET-minutes per week (MET min/week) for walking, moderate and vigorous activity, respectively (Craig et al. 2003). For descriptive purposes, participants were classified as high, moderate, or low according to IPAQ categorical criteria. Highly active was defined as 7 or more days of any combination of walking, moderate-intensity, or vigorous-intensity activities, achieving a minimum total physical activity of ≥ 3000 MET-min/week. Moderately active was defined as 5 or more days of any combination of walking, moderate-intensity, or vigorous-intensity activities, amounting to a minimum total physical activity of ≥ 600 MET-min/week. Participants were classified as low if they did not meet the criteria for the moderate category.

### Sarcopenia classification and muscle quality

Two established definitions were applied to the sample groups to establish the prevalence of sarcopenia. ALM was calculated as the sum of the fat-free mass in the limbs, excluding bone mineral content, divided by body mass and expressed as a percentage. This definition has been applied and revealed a 40.4% prevalence rate of sarcopenia (Mayhew et al. 2018). In the present study, cut-points of < 27.1% for males and < 22.3% for females were used to define sarcopenia.

The second definition was the composite measure of the International Working Group on Sarcopenia (IWGS) (Fielding et al. 2011), representing the lowest prevalence limit (9.9%) according to Mayhew et al. (2018). Participants with a gait speed of < 1 m/s were identified and subsequently screened by lean tissue mass. ALM/height^2^ was calculated and the cut-points of ≤ 7.23 kg/m^2^ for males and ≤ 5.67 kg/m^2^ for females were applied to determine sarcopenia.

Upper-body MQ was calculated by dividing the maximum handgrip strength by upper body ALM, with cut-points determined as < 5.76 kg/kg for males and < 5.475 kg/kg for females, respectively (Cooper et al. 2014). Lower-body MQ was determined as the ratio of dominant leg extension strength to leg lean mass (Hairi et al. 2010). Since to our knowledge cut-points for younger populations do not currently exist, cautious and limited comparisons of upper-body MQ for the younger group were made against established norms for the older group. Protocol-specific cut-points for lower-body MQ using the adopted MQ algorithm do not currently exist. Therefore, and in line with the selection of cut-points for sarcopenia (Baumgartner et al. 1998), low MQ in the lower body was classified as at least two standard deviations below the mean of the corresponding younger group in the present study, with cut-points of ≤ 2.11 kg/kg for males and ≤ 1.56 kg/kg for females, respectively.

### Statistical analysis

All statistical analyses were performed using IBM SPSS statistics software (Version 24.0, IBM Corp., Armonk, NY). Prior to analysis, assumptions of normality in the data were made using Shapiro–Wilk tests and visualisation of normality plots. Between-group differences were investigated using multivariate analysis of variance (MANOVA). Cohen’s *d* effect sizes were calculated to quantify the magnitude of differences in body composition, functional and strength testing, MQ, and estimates of physical activity. The effect size thresholds were classified in line with Cohen (1988): < 0.2 = trivial; 0.2–0.5 = small; 0.5–0.8 = medium; 0.8–1.3 = large; > 1.3 = very large.

The relationships between total ALM and strength, functional performance and physical activity were determined for each group using Pearson’s correlation coefficients. The strength of the relationship was classified according to the thresholds of Cohen (1988): small = 0.10, moderate = 0.30, large = 0.50. Fisher *r*-to-*z* transformations were performed to assess the significance of differences in correlation coefficients between groups.

By way of multiple regression analysis using the forced entry method, the best predictors of upper and lower body ALM were determined for both sample groups. Adjusted *R*^2^ and multicollinearity statistics (tolerance and variance inflation factor) were used to identify the optimum equations (Krause et al. 2012; Stoever et al. 2017). For all regression models, dominant handgrip strength and dominant leg extension strength were chosen as the best predictors. For all appropriate analyses, 95% confidence intervals (95% CI) are reported. All data are presented as mean ± standard deviation (SD) with statistical significance for all analyses set at *P* ≤ 0.05.

## Results

### Basic anthropometry and preliminary screening

Comparisons of basic anthropometry and preliminary screening measures are given in Table 1. The younger group was significantly taller and had lower resting systolic blood pressure than the older group. No significant differences were found for body mass, BMI, resting heart rate, or resting diastolic blood pressure. Blood lipid profile components and blood pressure in both groups were within established norms for metabolically healthy individuals (data not shown).

### Body composition assessment

Comparisons of body composition, functional measures and strength testing are presented in Table 2. The younger group had significantly lower fat mass and %TFM than the older group. Lean mass and total ALM were both significantly higher in the younger group.

**Table 2:** Body composition, functional performance, physical activity and strength testing measures by age group (*n* = 100)

Variable	Young (*n* = 50)	Older (*n* = 50)	*P* value	Effect size (95% CI)
**Body composition**				
Fat mass (kg)	17.5 ± 6.5	22.6 ± 7.6	0.001	0.72 (0.30, 1.10)
Lean mass (kg)	54.2 ± 11.1	45.0 ± 10.4	< 0.0001	0.85 (0.38, 1.30)
%TFM	24.5 ± 8.4	32.7 ± 7.4	< 0.0001	1.04 (0.64, 1.40)
Total-body ALM (kg)	25.4 ± 6.0	20.1 ± 5.0	< 0.0001	0.96 (0.56, 1.40)
Upper-body ALM (kg)	6.7 ± 2.3	5.0 ± 1.5	< 0.0001	0.88 (0.49, 1.30)
Lower-body ALM (kg)	18.6 ± 3.9	15.1 ± 3.6	< 0.0001	0.93 (0.54, 1.30)
**Strength, muscle quality and functional performance**				
Dominant HGS (kg)	40.4 ± 10.5	29.1 ± 8.5	< 0.0001	1.18 (0.78, 1.60)
Non-dominant HGS (kg)	38.6 ± 10.9	27.3 ± 8.9	< 0.0001	1.13 (0.74, 1.70)
Dominant leg extension 1RM (kg)	58.3 ± 21.4	27.9 ± 8.6	< 0.0001	1.86 (1.46, 2.30)
Non-dominant leg extension 1RM (kg)	56.8 ± 20.1	26.3 ± 8.8	< 0.0001	1.97 (1.57, 2.40)
Upper-body MQ (kg/kg)	6.3 ± 1.3	5.9 ± 1.1	0.136	0.33 (− 0.11, 0.77)
Lower-body MQ (kg/kg)	3.1 ± 0.7	1.9 ± 0.4	< 0.0001	2.10 (1.72, 2.50)
6-m gait speed (m/s)	1.34 ± 0.17	1.21 ± 0.24	0.002	0.63 (0.24, 1.00)
**Physical activity**				
Walking (MET-min/week)	2276 ± 2254	1933 ± 1713	0.393	0.17 (− 0.22, 1.56)
Moderate (MET-min/week)	1537 ± 2068	2124 ± 2393	0.193	0.26 (− 0.13, 0.65)
Vigorous (MET-min/week)	3266 ± 2815	1642 ± 3131	0.008	0.63 (0.17, 1.10)
Total (MET-min/week)	7079 ± 4868	5699 ± 5045	0.167	0.27 (− 0.11, 0.65)

Data presented as mean ± SD

*%TFM* percentage tissue fat mass, *1RM* one repetition maximum, *ALM* appendicular lean mass, *HGS* handgrip strength, *MET* metabolic equivalent, *MQ* muscle quality

In both groups, lean mass was strongly and significantly associated with total ALM (Table 3). In the older group only, there was a moderate positive correlation between total ALM and fat mass. In the younger group, fat mass was negatively associated with lean mass (*r* = -0.22), however this was not statistically significant (*P* = 0.130). In the older group, fat mass was positively associated with lean mass (*r* = 0.53; *P* < 0.0001). All correlation coefficients were significantly different between groups.

**Table 3:** Correlates of total appendicular lean mass by age group. Fisher *r*-to-*z* transformations were performed to assess the significance of between-group differences in correlation coefficients

Variable	Young (*n* = 50)	Older (*n* = 50)	*Z* score	*P* value
**Body composition**				
Fat mass (kg)	− 0.16	0.45**	− 3.13	0.002
Lean mass (kg)	0.99**	0.88**	6.16	< 0.001
%TFM	− 0.56**	− 0.21	− 2.03	0.042
**Strength, muscle quality and functional performance**				
Dominant HGS (kg)	0.78**	0.79**	− 0.13	0.897
Non-dominant HGS (kg)	0.78**	0.79**	− 0.13	0.897
Dominant leg extension 1RM (kg)	0.87**	0.71**	2.16	0.031
Non-dominant leg extension 1RM (kg)	0.83**	0.74**	1.15	0.250
Upper-body MQ (kg/kg)	− 0.61**	− 0.31*	− 1.88	0.060
Lower-body MQ (kg/kg)	0.51**	− 0.04	2.92	0.004
6-m gait speed (m/s)	− 0.14	0.00	− 0.68	0.497
**Physical activity**				
Walking (min/week)	− 0.03	− 0.17	0.69	0.490
Moderate (min/week)	− 0.13	− 0.21	0.40	0.689
Vigorous (min/week)	0.08	0.18	− 0.49	0.624
Total (MET-min/week)	− 0.03	− 0.05	0.01	0.920

*%TFM* percentage tissue fat mass, *1RM* one repetition maximum, *ALM* appendicular lean mass, *HGS* handgrip strength, *MET* metabolic equivalent, *MQ* muscle quality

*Significant at *P* < 0.05; **significant at *P* < 0.01

### Functional measures and strength testing

Upper and lower-body strength testing revealed significantly greater strength in the younger group (Table 2). No significant differences were found in upper-body MQ, whereas lower-body MQ was significantly greater in the younger group. Gait speed was significantly faster in the younger group.

In both groups, large and significant positive associations were found between upper and lower body strength and total ALM (Table 3). Negative associations were found between upper-body MQ and total ALM in both groups. In addition, a large and significant positive association was found between lower-body MQ and total ALM in the younger group. None of the coefficients differed significantly between groups except for dominant leg extension strength and lower-body MQ.

### Assessment of habitual physical activity

The younger group demonstrated significantly greater levels of vigorous activity than the older group. All other comparisons were not significantly different between groups. No significant associations were found between total ALM and IPAQ-derived data in either group, and the correlation coefficients did not differ significantly between groups.

Using the IPAQ categorical classification criteria, the younger group was profiled as follows: low = 2, moderate = 7, high = 41. The older group was as follows: low = 1, moderate = 16, high = 33.

### Sarcopenia classification and muscle quality status

As per the definitions put forward by the IWGS, one older male was classified as sarcopenic. When sarcopenia was calculated using lean tissue mass as the sole measure, derived from ALM/body mass, only two older males were classified as sarcopenic.

Upper-body MQ was below established cut-points in 16 young (14 males, 2 females) and 21 older (14 males, 7 females) participants. For lower-body MQ, 17 older males and 8 older females were at least 2 standard deviations below the mean of the corresponding younger group. Nine older males had low MQ at both the upper and lower body, whereas this was only the case in two older females.

### Multiple regression analyses

The results of the multiple regression models for the younger group are shown in Table 4. These models accounted for 82% and 74% of the variance in upper and lower body ALM, respectively, using dominant handgrip strength and dominant leg extension strength as predictor variables. The complete equations are as follows:

**Table 4:** Results of the multiple regression models for the prediction of upper and lower body ALM in young individuals (*n* = 50)

Model	Unstandardised coefficients		Standardised coefficients	*P* value	Collinearity statistics	
	*B* (95% CI)	SE	Beta		Tolerance	VIF
**Upper body ALM**						
Constant	− 0.310 (− 1.436, 0.817)	0.560		0.583		
Dominant leg 1RM	0.065 (0.046, 0.083)	0.009	0.607	< 0.00001	0.518	1.930
Dominant HGS	0.081 (0.044, 0.119)	0.019	0.373	< 0.0001	0.518	1.930
**Lower body ALM**						
Constant	7.420 (5.119, 9.720)	1.143		< 0.00001		
Dominant leg 1RM	0.111 (0.074, 0.148)	0.019	0.613	< 0.00001	0.518	1.930
Dominant HGS	0.117 (0.040, 0.193)	0.038	0.315	0.004	0.518	1.930

*R* = 0.91 and *R*^2^ = 0.82 for upper body regression model; *R* = 0.86 and *R*^2^ = 0.74 for lower body regression model

*1RM* one-repetition maximum, *ALM* appendicular lean mass, *HGS* handgrip strength, *VIF* variance inflation factor

$${\text{Upper}}\;{\text{body}}\;{\text{ALM}}= - 0.310+0.065{\text{ }}{x_{{\text{dominant}}\;{\text{leg}}\;{\text{extension}}\;1{\text{RM}}}}+{\text{ }}0.081{\text{ }}{x_{{\text{dominant}}\;{\text{grip}}\;{\text{strength}}}}$$

$${\text{Lower}}\;{\text{body}}\;{\text{ALM}}=7.420+0.111{\text{ }}{x_{{\text{dominant}}\;{\text{leg}}\;{\text{extension}}\;1{\text{RM}}}}+0.117{\text{ }}{x_{{\text{dominant}}\;{\text{grip}}\;{\text{strength}}}}$$

The same multiple regression models were applied to the older group and are shown in Table 5. The models accounted for 72% and 68% of the variance in upper and lower body ALM, using the same respective predictors. The complete equations are as follows:

**Table 5:** Results of the multiple regression models for the prediction of upper and lower body ALM in older individuals (*n* = 50)

Model	Unstandardised coefficients		Standardised coefficients	*P* value	Collinearity statistics	
	*B* (95% CI)	SE	Beta		Tolerance	VIF
**Upper body ALM**						
Constant	0.330 (− 0.561, 1.220)	0.443		0.460		
Dominant leg 1RM	0.072 (0.039, 0.104)	0.016	0.418	< 0.0005	0.655	1.526
Dominant HGS	0.093 (0.059, 0.126)	0.017	0.533	< 0.00001	0.655	1.526
**Lower body ALM**						
Constant	3.865 (1.542, 6.188)	1.155		0.002		
Dominant leg 1RM	0.147 (0.061, 0.233)	0.043	0.350	0.001	0.655	1.526
Dominant HGS	0.244 (0.157, 0.331)	0.043	0.571	< 0.00001	0.655	1.526

*R* = 0.85 and *R*^2^ = 0.72 for upper body regression model; *R* = 0.83 and *R*^2^ = 0.68 for lower body regression model

*1RM* one-repetition maximum, *ALM* appendicular lean mass, *HGS* handgrip strength, *VIF* variance inflation factor

$${\text{Upper}}\;{\text{body}}\;{\text{ALM}}=0.330+0.072{x_{{\text{dominant}}\;{\text{leg}}\;{\text{extension}}\;1{\text{RM}}}}+0.093{x_{{\text{dominant}}\;{\text{grip}}\;{\text{strength}}}}$$

$${\text{Lower}}\;{\text{body}}\;{\text{ALM}}=3.865+0.147{x_{{\text{dominant}}\;{\text{leg}}\;{\text{extension}}\;1{\text{RM}}}}+{\text{ }}0.244{x_{{\text{dominant}}\;{\text{grip}}\;{\text{strength}}}}$$

## Discussion

The aims of the present pilot study were to investigate differences in lean tissue mass, functional capacity and physical activity between younger and older individuals and to establish relationships between these variables and total ALM. Muscle quality assessment was performed at the upper and lower body, and these findings were reconciled with two established definitions of sarcopenia. Finally, regression analyses were performed to investigate the application of upper and lower body ALM for use as a proxy measure of MQ. The major findings of this study suggest that in spite of low sarcopenia rates (2–4% using two established definitions) in our older population, 42% had low upper-body MQ, as per existing thresholds. Interestingly, 32% of the younger group were also below these thresholds. In spite of the absence of protocol-specific benchmarks for lower-body MQ, we found that 50% of the older group had MQ values at least two standard deviations below the mean for the respective younger groups.

Our findings show that the younger group possessed lower fat mass and %TFM than the older group. Greater lean mass and ALM were also found in the younger group, with all comparisons supported by large effect sizes. These observations corroborate those of previous studies, suggesting that fat mass increases and lean mass decreases with age, without substantial differences in body mass (St-Onge and Gallagher 2010; Mazariegos et al. 1994; Atlantis et al. 2008).

Fat mass was positively correlated with both total ALM and lean mass in the older group only, and these findings concur with previous studies (Bouchard et al. 2011; Lebrun et al. 2006). This relationship may be important for clinical outcomes. Low fat mass and lean tissue mass pose the greatest risk of all-cause mortality in older individuals (Spahillari et al. 2016). Conversely, greater lean tissue mass is associated with improved overall mortality in older individuals (Spahillari et al. 2016). Greater fat mass is associated with a decreased risk of adverse events in hospitalised elderly patients (Bouillanne et al. 2009). Additionally, the overweight classification for the older group in the present study is associated with the lowest mortality rate in this population (Chang et al. 2012; Kvamme et al. 2012; Bouillanne et al. 2009). Together, these findings support the notion that lean tissue mass improvements should be emphasised in older adults ahead of measures such as BMI (Spahillari et al. 2016).

In the present study, maximal strength at the upper and lower body was significantly greater in the young, constituting large and very large effects, respectively. These findings are in line with previous literature that showed greater torque, normalised torque and power in younger versus older men, with the lower body affected more substantially than the upper body (Candow and Chilibeck 2005). Gait speed was significantly lower in the older group (moderate effect), and our findings correspond with Laufer (2005), who observed reduced forward walking velocity in older compared to younger individuals.

Upper-body MQ did not differ significantly between groups (small effect). In the older group, upper-body MQ was similar to that observed by Hairi et al. (2010), albeit in the 80–84 year old age group. Low upper-body MQ was prevalent in 42% of the older group, and in 32% of the younger group based on established cut-points (Cooper et al. 2014). The latter finding should be treated with caution since the aforementioned cut-points are age-specific. Nevertheless, this emphasises the need for further work to develop and establish age-specific MQ cut-points. The negative associations between total ALM and upper-body MQ in both groups are explained as a function of the MQ equation, in that the denominator (total ALM) includes tissue (i.e., lower body ALM) that has no impact on force production during handgrip strength assessment.

Lower-body MQ was significantly greater in the young (very large effect). It must also be noted that no protocol-specific cut-point thresholds have been defined for lower-body MQ metrics in young and older individuals, and this represents an avenue for future research. In contrast with upper-body MQ, lower-body MQ was positively associated with total ALM in the young; however, no effect was found for this variable in the older group. The absence of an association between total ALM and lower-body MQ in the older group warrants further exploration. Muscle quality has been shown to decline progressively with age, potentially as a result of neurological factors, such as decrements in excitation contraction coupling and motor nerve conduction velocity (Moore et al. 2014; Clark and Manini 2012). Therefore, our findings suggest that measures of lean mass and sarcopenia definitions cannot solely be used to determine musculoskeletal health in young and older adults, and reinforce the rationale for MQ assessment of the upper and lower body (Cruz-Jentoft et al. 2018).

Self-reported physical activity was significantly higher in the younger group for vigorous activities (moderate effect), whereas all other comparisons were non-significant between groups. No significant associations were found between total ALM and IPAQ-derived data in each group and the correlation coefficients did not significantly differ. These findings lend further support to the use of strength testing to predict ALM, whilst demonstrating that IPAQ data cannot be used to predict ALM.

In totality, our findings lend support for the assessment of lower-body strength testing and reinforce the importance of this metric as a key predictor of physical performance and function (Marsh et al. 2006; Coelho-Junior et al. 2018; Yeung et al. 2018; Bouchard et al. 2011). The use of handgrip strength testing is ubiquitous in the literature (Bischoff-Ferrari et al. 2015; Newman et al. 2003, 2006; Stoever et al. 2017; Lauretani et al. 2003), given its use as a highly efficient screening tool (Martin et al. 2006). Despite this, handgrip strength may misclassify individuals as it only accounts for ~ 40% of the variance in lower body strength (Manini and Clark 2012). Moreover, caution should be observed when characterising overall strength based on the use of a single measurement tool (Bohannon 2008; Mitchell et al. 2012; Tieland et al. 2015). In older adults, differential associations have been noted between health characteristics, knee extension strength and handgrip strength, to the extent that lower-body strength testing poses considerable additive value (Yeung et al. 2018). Therefore, we support the inclusion of lower-body strength testing in addition to that of handgrip strength to enable better prediction of ALM in older adults. The measurement of leg extension strength using cost-effective and practical equipment appears to be a useful alternative to isokinetic dynamometry, and aligns well with recent recommendations concerning strength testing in older adults (Cruz-Jentoft et al. 2018).

In conclusion, an important finding of the present study was the low number of older individuals identified as sarcopenic, and the similarity of two established definitions in detecting these individuals despite large pooled prevalence differences between definitions (Mayhew et al. 2018). Deficits in upper-body MQ were found in the younger (*n* = 16) and older (*n* = 21) groups. Furthermore, 25 older individuals had low MQ at the lower body. Eleven of the older individuals had low ‘global’ MQ (i.e., upper and lower), representing 22% of the older group. These findings lend support to the rationale for conducting MQ testing alongside established sarcopenia definitions to effectively identify individuals at risk of functional decline. Future studies should seek to investigate the veracity of our regression models in a larger and more diverse sample population. In this manner, MQ at the upper and lower body might be estimated using strength measures and implemented as a practical prognostic tool. Individuals with low ‘estimated’ MQ may then be referred for DXA imaging as necessary to confirm or deny the presence of sarcopenia and/or impaired MQ.

## Abbreviations

%TFM: Percentage tissue fat mass
1RM: One-repetition maximum
ALM: Appendicular lean mass
BMI: Body mass index
DBP: Diastolic blood pressure
DXA: Dual-energy X-ray absorptiometry
HGS: Handgrip strength
IPAQ: International Physical Activity Questionnaire
IWGS: International Working Group on Sarcopenia
MANOVA: Multivariate analysis of variance
MET: Metabolic equivalent
MQ: Muscle quality
RHR: Resting heart rate
SBP: Systolic blood pressure
SD: Standard deviation
VIF: Variance inflation factor
